# Enhancement of TRAIL-induced apoptosis by 5-fluorouracil requires activating Bax and p53 pathways in TRAIL-resistant lung cancers

**DOI:** 10.18632/oncotarget.14994

**Published:** 2017-02-02

**Authors:** Uddin MD. Nazim, Mohammad Rasheduzzaman, You-Jin Lee, Dai-Wu Seol, Sang-Youel Park

**Affiliations:** ^1^ Biosafety Research Institute, College of Veterinary Medicine, Chonbuk National University, Iksan, Jeonbuk 54596, Republic of Korea; ^2^ Faculty of Pharmacy, Chung-Ang University School of Pharmacy, Seoul 156-756, Republic of Korea

**Keywords:** 5-fluorouracil, TRAIL, apoptosis, lung cancer cells

## Abstract

Lung cancer, especially lung adenocarcinoma, is one of the main causes of death worldwide. Tumor necrosis factor-related apoptosis-inducing ligand (TRAIL) is a primary anticancer agent and a member of the tumor necrosis factor family that selectively induces apoptosis in various tumor cells, but not in normal cells. Combination chemotherapy can be used for treating specific cancer types even at progressive stages. In the present study, we observed that 5-fluorouracil, which exerts anticancer effects by inhibiting tumor cell proliferation, enhanced TRAIL-induced apoptosis of TRAIL-resistant human adenocarcinoma A549 cells. Interestingly, 5-fluorouracil treatment markedly increased Bax and p53 levels and 5-fluorouracil and TRAIL cotreatment increased Ac-cas3 and Ac-cas8 levels compared with those in control cells. Taken together, the present study demonstrated that 5-fluorouracil enhances TRAIL-induced apoptosis in TRAIL-resistant lung adenocarcinoma cells by activating Bax and p53, and also suggest that TRAIL and 5-fluorouracil cotreatment can be used as an adequate therapeutic strategy for TRAIL-resistant human cancers.

## INTRODUCTION

Lung cancer, especially lung adenocarcinoma, is one of the main causes of death worldwide [1]. Approximately 85% patients with lung cancer have non-small cell lung cancer (NSCLC), a histologically heterogeneous type of lung cancer [2, 3]. Cytotoxic chemotherapy moderately prolongs continuity for patients with advanced NSCLC. Multiple options are available for treating patients with lung cancer, including radiation therapy, chemotherapy, surgery, and its combinations [4, 5]. Combination chemotherapy can be used for treating specific cancer types even at progressive stages. Use of different drug combinations has played an important role in cancer treatment over several years.

Tumor necrosis factor (TNF)-related apoptosis-inducing ligand (TRAIL) is a typical member of the TNF superfamily [6, 7] that plays a potential role in the proliferation, differentiation, and apoptosis of tumor cells [8]. TRAIL activation induces apoptotic pathway by stimulating TRAIL receptors (TRAIL-R1 and TRAIL-R2; also known as death receptors 4 and 5 [DR4 and DR5], respectively) on the surface of target cells [9]. Other three receptors, namely, TRAIL-R3 (DcR1), TRAIL-R4 (DcR2; also called “decoy receptors”), and osteoprotegerin/TRAIL-R5, cannot trigger the apoptotic cascade because of the absence of functional death domains [10]. Binding of TRAIL to the death receptors DR4 and DR5 recruits Fas-associated death domain protein and eventually procaspase-8 to form a death-inducing signaling complex on the inner surface of the plasma membrane of cancer cells, which in turn leads to caspase-8 activation [11, 12]. Active caspase-8 induces intrinsic and extrinsic apoptotic pathways by activating downstream caspases such as caspase-3 or by cleaving Bid, a member of Bcl-2 family [13].

5-Fluorouracil is a pyrimidine analogue that was first developed in 1957 [14]. 5-Fluorouracil exerts anticancer effects by inhibiting thymidylate synthase and by disrupting DNA synthesis and induces the apoptosis of cancer cells by affecting uracil metabolism [15]. Recent studies indicate that 5-fluorouracil, which increases the apoptosis of cancer cells, has been widely used for treating liver [16], renal [17], breast [18], and gastric [19] cancers.

Apoptosis or type I programmed cell death is morphologically characterized by cell shrinkage, chromatin condensation, DNA fragmentation, and distinct apoptotic body formation [20]. Apoptosis is induced by the intrinsic apoptotic pathway, which is usually activated by endogenous stresses such as DNA damage, hypoxia, or other cellular stresses, and the extrinsic apoptotic pathway, which is induced by cell surface death receptors such as TNF receptor superfamily [21, 22]. Tumor suppressor protein p53 induces apoptosis by coordinating with several cellular components. Bax protein is a purpose of p53 transcription factor activity and Bax induction has been noticed during p53-mediated apoptosis [23, 24]. In cancer cells, activation of the apoptotic pathway plays a major protective role against cancer progression.

Several anticancer chemotherapeutic drugs such as TRAIL induce apoptosis and may participate in common intracellular signaling pathways leading to the apoptosis of cancer cells. We observed that TRAIL and 5-fluorouracil cotreatment sensitized TRAIL-resistant human lung adenocarcinoma A549 cells. Thus, we investigated molecular mechanisms underlying the anticancer effect of 5-fluorouracil and the synergistic effect of 5-fluorouracil and TRAIL cotreatment on TRAIL-resistant A549 cells.

## RESULTS

### 5-Fluorouracil enhances TRAIL-induced apoptosis of lung adenocarcinoma cells

**Figure 1:**
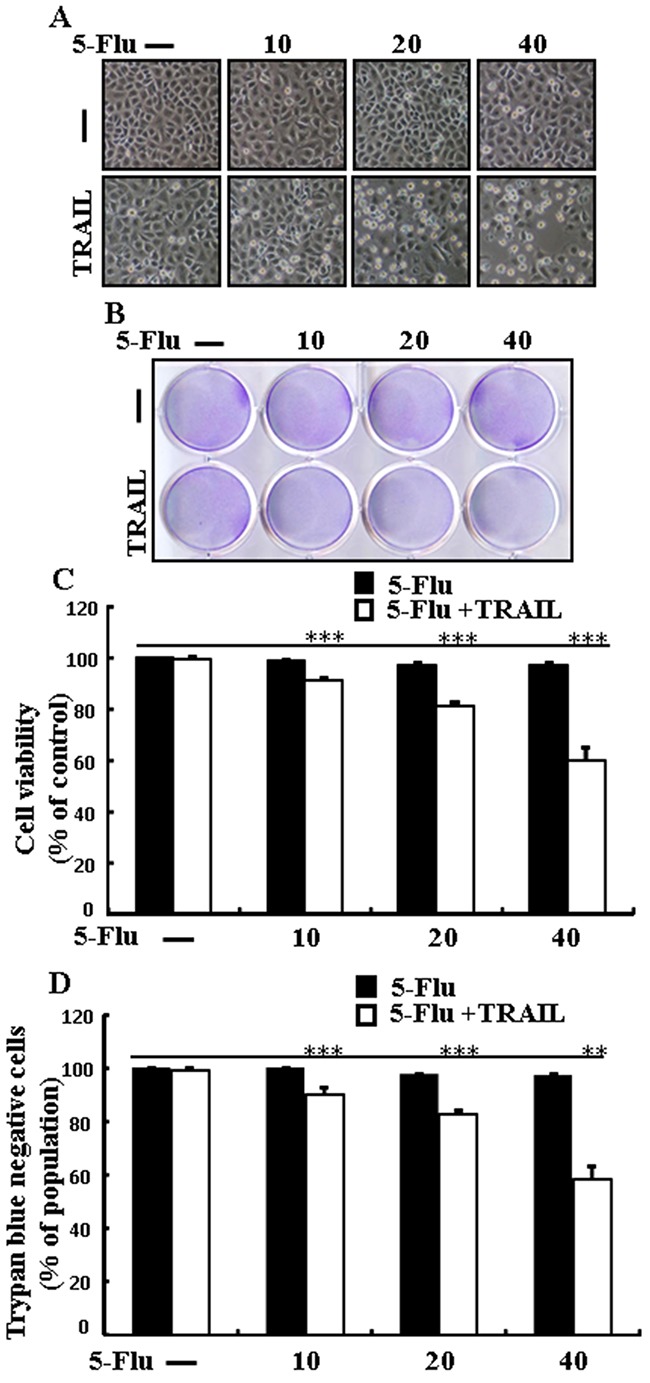
5-Fluorouracil enhances TRAIL-induced apoptosis in lung adenocarcinoma cells A549 cells were pretreated with 5-fluorouracil at varying concentrations (0, 10, 20, and 40 μM) for 12 h followed by treatment with 200 ng/mL of TRAIL protein for an additional 2 h. **A**. Cell morphology photographed under light microscope in A549 Cells (×100); **B**. Cell viability determined with crystal violet assay in A549 Cells; **C**. Bar graph showing the average density of crystal violet dye in A549 Cells; **D**. Cell viability determined with trypan blue dye exclusion assays in A549 Cells. ** *p* < 0.01, *** *p* < 0.001: significant differences between control and each treatment group; 5-Flu: 5-Fluorouracil; TRAIL: Tumor necrosis factor (TNF)-related apoptosis-inducing ligand.

To determine the effect of 5-fluorouracil on TRAIL-induced apoptosis, A549 cells were pretreated with different concentrations of 5-fluorouracil for 12 h, followed by treatment with TRAIL for 2 h. Changes in cell morphology were determined by photographing the cells under a light microscope. 5-Fluorouracil or TRAIL treatment alone did not or only slightly affected cell viability (Figure 1) and did not induce morphological changes compared with that in control cells, indicating that A549 cells were highly resistant to TRAIL. However, cotreatment with TRAIL and different concentrations of 5-fluorouracil significantly decreased cell viability compared with 5-fluorouracil or TRAIL treatment alone. Cell morphology results also revealed this enhanced effect of 5-fluorouracil, showing that the combination of TRAIL and 5-fluorouracil enhanced the number of apoptotic cell deaths compared with treatment with 5-fluorouracil or TRAIL alone (Figure 1A). Furthermore, TRAIL and 5-fluorouracil cotreatment decreased the viability and significantly increased the apoptosis of A549 cells (Figure 1B, 1C, and 1D). These results indicate that 5-fluorouracil significantly increases TRAIL-induced apoptosis of TRAIL-resistant human lung adenocarcinoma A549 cells.

### Effects of 5-fluorouracil on death receptors and enhances apoptosis mediated by TRAIL

**Figure 2:**
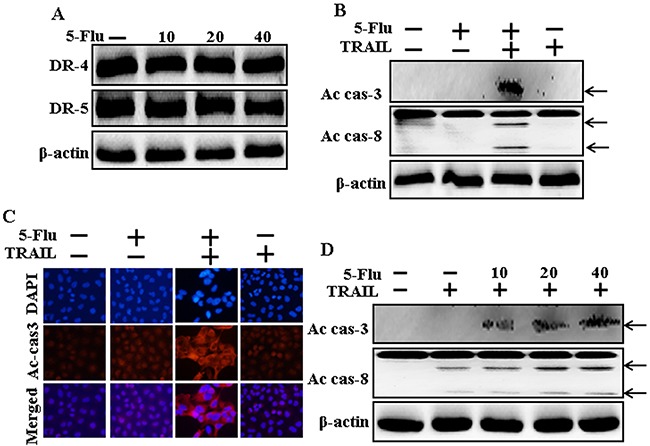
Effects of 5-fluorouracil on death receptor and enhances apoptosis mediated by TRAIL A549 adenocarcinoma cells were pretreated with 5-fluorouracil at different concentrations (0, 10, 20, and 40 μM) for 12 h. **A** and **B**. Cells were harvested and analyzed by Western blotting to determine the expression levels of DR-4, DR-5, Ac-cas3 and Ac-cas8; **C**. Representative immunocytochemistry was implemented in A549 cells after co-treatment with 5-fluorouracil (12 h) and 200 ng/ml TRAIL (1 h); **D**. Ac-cas3 and Ac-cas8 expression levels determined by western blot analysis. β-actin was used as loading control. 5-Flu: 5-Fluorouracil; Ac-cas3: Activated caspase 3; Ac-cas8: Activated caspase 8; TRAIL: Tumor necrosis factor (TNF)-related apoptosis-inducing ligand.

To determine the effect of 5-fluorouracil on the death receptors, lung adenocarcinoma A549 cells were pretreated with serial concentrations of 5-fluorouracil for 12 h, followed by treatment with TRAIL for 1 h. Whole-cell lysates were obtained and were subjected to western blotting analysis. Results of western blotting showed that levels of TRAIL receptors DR4 and DR5 were unchanged in cells treated with the serial concentrations of 5-fluorouracil (Figure 2A). However, A549 cells cotreated with TRAIL and 5-fluorouracil showed higher Ac-cas3 and Ac-cas8 expression than cells treated with 5-fluorouracil or TRAIL alone (Figure 2B). Results of immunocytochemistry (ICC) also showed that TRAIL and 5-fluorouracil cotreatment increased Ac-cas3 expression (Figure 2C). Moreover, our results showed that TRAIL and 5-fluorouracil cotreatment increased Ac-cas3 and Ac-cas8 expression gradually (Figure 2D). These results suggest that 5-fluorouracil enhances the apoptosis of TRAIL-resistant human lung adenocarcinoma A549 cells.

### 5-Fluorouracil enhances TRAIL-mediated Bax expression in A549 cells

**Figure 3:**
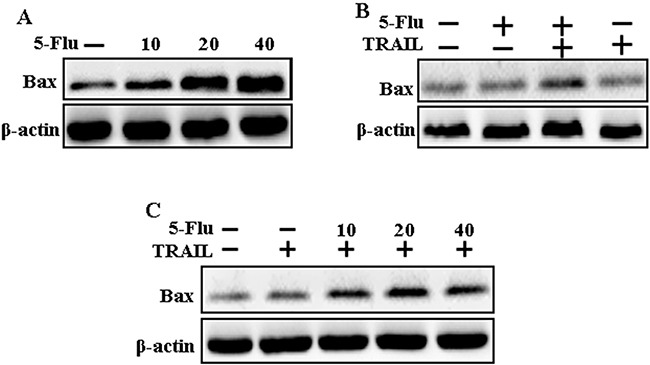
5-Fluorouracil enhanced Bax expression in A549 cells mediated by TRAIL A549 adenocarcinoma cells were pretreated with 5-fluorouracil at different concentrations (0, 10, 20, and 40 μM) for 12 h. **A**. Cells were harvested and analyzed by Western blotting to determine the expression levels of Bax; **B** and **C**. Bax expression levels determined by western blot analysis. A549 cells were pre-treated with 5-fluorouracil for 12 h and then exposed to 200 ng/mL TRAIL for an additional 1 h. β-actin was used as loading control. 5-Flu: 5-Fluorouracil; Ac-cas3: Activated caspase 3; Ac-cas8: Activated caspase 8; TRAIL: Tumor necrosis factor (TNF)-related apoptosis-inducing ligand.

To determine the effect of 5-fluorouracil on Bax expression, lung adenocarcinoma A549 cells were pretreated with serial concentrations of 5-fluorouracil for 12 h, followed by treatment with TRAIL for 1 h. Whole-cell lysates were obtained and were subjected to western blotting analysis. Results of western blotting showed that 5-fluorouracil treatment increased Bax expression in a dose-dependent manner (Figure 3A). However, Bax expression was higher in cells cotreated with TRAIL and 5-fluorouracil than in cells treated with 5-fluorouracil or TRAIL alone (Figure 3B). Moreover, TRAIL and 5-fluorouracil cotreatment gradually increased Bax expression in A549 cells (Figure 3C). These results suggest that 5-fluorouracil induces Bax expression in TRAIL-resistant human lung adenocarcinoma A549 cells.

### Effects of 5-fluorouracil in Bax-containing (Bax^+/+^) and Bax-deficient (Bax^−/−^) HCT116 human colon carcinoma cells mediated by TRAIL

**Figure 4:**
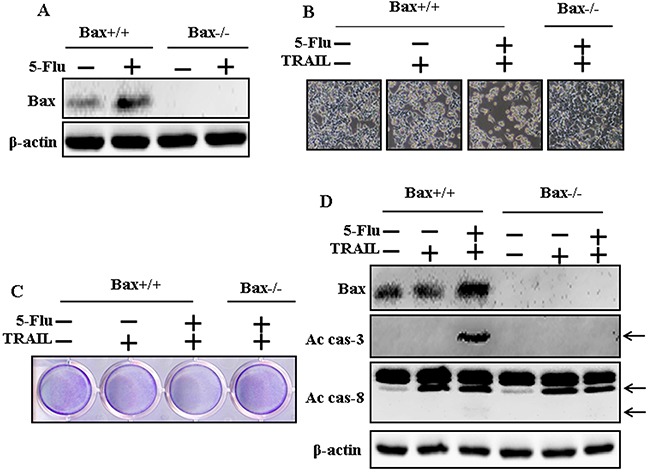
Effects of 5-fluorouracil in Bax-containing (Bax+/+) and Bax-deficient (Bax−/−) HCT116 human colon carcinoma cells mediated by TRAIL Bax-containing (Bax+/+) and Bax-deficient (Bax−/−) HCT116 human colon carcinoma cells were pretreated with 5-fluorouracil (40 μM) for 12 h. After that, cells were treated with 200 ng/mL of TRAIL protein for an additional 2 h. **A**. Cells were harvested and analyzed by Western blotting to determine the expression levels of Bax; **B**. Cell morphology photographed under light microscope (×100); **C**. Cell viability determined with crystal violet assay; **D**. Bax, Ac-cas3 and Ac-cas8 expression levels determined by western blot analysis. β-actin was used as loading control. 5-Flu: 5-Fluorouracil; Ac-cas3: Activated caspase 3; Ac-cas8: Activated caspase 8; TRAIL: Tumor necrosis factor (TNF)-related apoptosis-inducing ligand.

To determine the effect of 5-fluorouracil on TRAIL-induced apoptosis, HCT116 human colon carcinoma cells were pretreated with 5-fluorouracil (40 μM) for 12 h, followed by treatment with TRAIL for 2 h. Whole-cell lysates were obtained and were subjected to western blotting analysis to determine changes in Bax, Ac-cas3, and Ac-cas8 expression levels. Bax expression levels increased after 5-fluorouracil treatment in Bax-containing (Bax^+/+^) cells but not in Bax-deficient (Bax^−/−^) cells (Figure 4A). Morphology and cell viability analyses showed that 5-fluorouracil and TRAIL cotreatment decreased the viability and significantly increased the apoptosis of Bax^+/+^ cells but not of Bax^−/−^ cells compared with TRAIL treatment alone (Figure 4B and 4C). Moreover, TRAIL and 5-fluorouracil cotreatment increased Bax, Ac-cas3, and Ac-cas8 expression levels in Bax^+/+^ cells but not in Bax^−/−^ cells (Figure 4D).

### 5-Fluorouracil enhanced p53 expression in A549, Bax-containing (Bax^+/+^) and Bax-deficient (Bax^−/−^) HCT116 human colon carcinoma cells mediated by TRAIL

**Figure 5:**
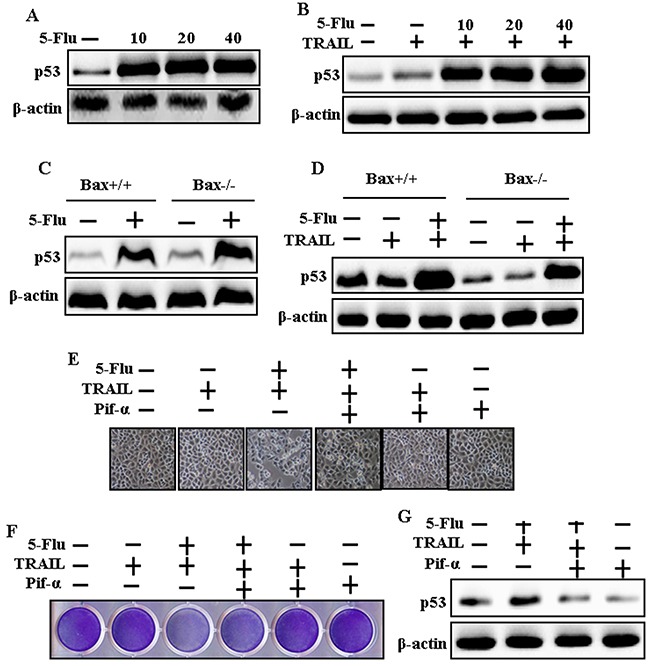
5-Fluorouracil enhanced p53 expression in A549, Bax-containing (Bax+/+) and Bax-deficient (Bax−/−) HCT116 human colon carcinoma cells mediated by TRAIL A549, Bax-containing (Bax+/+) and Bax-deficient (Bax−/−) HCT116 human colon carcinoma cells were pretreated with 5-fluorouracil at different concentrations (0, 10, 20, and 40 μM) for 12 h. A549 adenocarcinoma cells were also pretreated with Pifithrin-α for 1 h followed by treatment with 5-Fluorouracil (40 μM) for 12 h. After that, cells were treated with 200 ng/mL of TRAIL protein for an additional 2 h. **A, B, C, D** and **G**. p53 expression levels determined by western blot analysis. β-actin was used as loading control. **E**. Cell morphology photographed under light microscope in A549 Cells (×100); **F**. Cell viability determined with crystal violet assay in A549 Cells; 5-Flu: 5-Fluorouracil; Pif-α: Pifithrin-α; TRAIL: Tumor necrosis factor (TNF)-related apoptosis-inducing ligand.

To determine the effect of 5-fluorouracil on p53 expression, cells were pretreated with serial concentrations of 5-fluorouracil for 12 h, followed by treatment with TRAIL for 1 h. Whole-cell lysates were obtained and were subjected to western blotting analysis. 5-Fluorouracil treatment increased p53 expression in A549 cells in a dose-dependent manner (Figure 5A). However, TRAIL and 5-fluorouracil cotreatment increased p53 expression in A549 cells gradually (Figure 5B). 5-Fluorouracil treatment also increased p53 expression in Bax^+/+^ and Bax^−/−^ HCT116 human colon carcinoma cells (Figure 5C). Moreover, Bax^+/+^ and Bax^−/−^ HCT116 human colon carcinoma cells cotreated with TRAIL and 5-fluorouracil showed higher p53 expression than Bax^+/+^ and Bax^−/−^ HCT116 human colon carcinoma cells treated with TRAIL alone (Figure 5D). Morphological, crystal violet staining and western blot results show that co-treatment with 5-fluorouracil, TRAIL, and Pifithrin-α blocked the cell death effect compared with treatment with 5-fluorouracil and TRAIL in A549 lung cancer cells (Figure 5E, 5F, and 5G).

### 5-Fluorouracil enhances TRAIL-induced apoptosis of different lung cancer cell types

**Figure 6:**
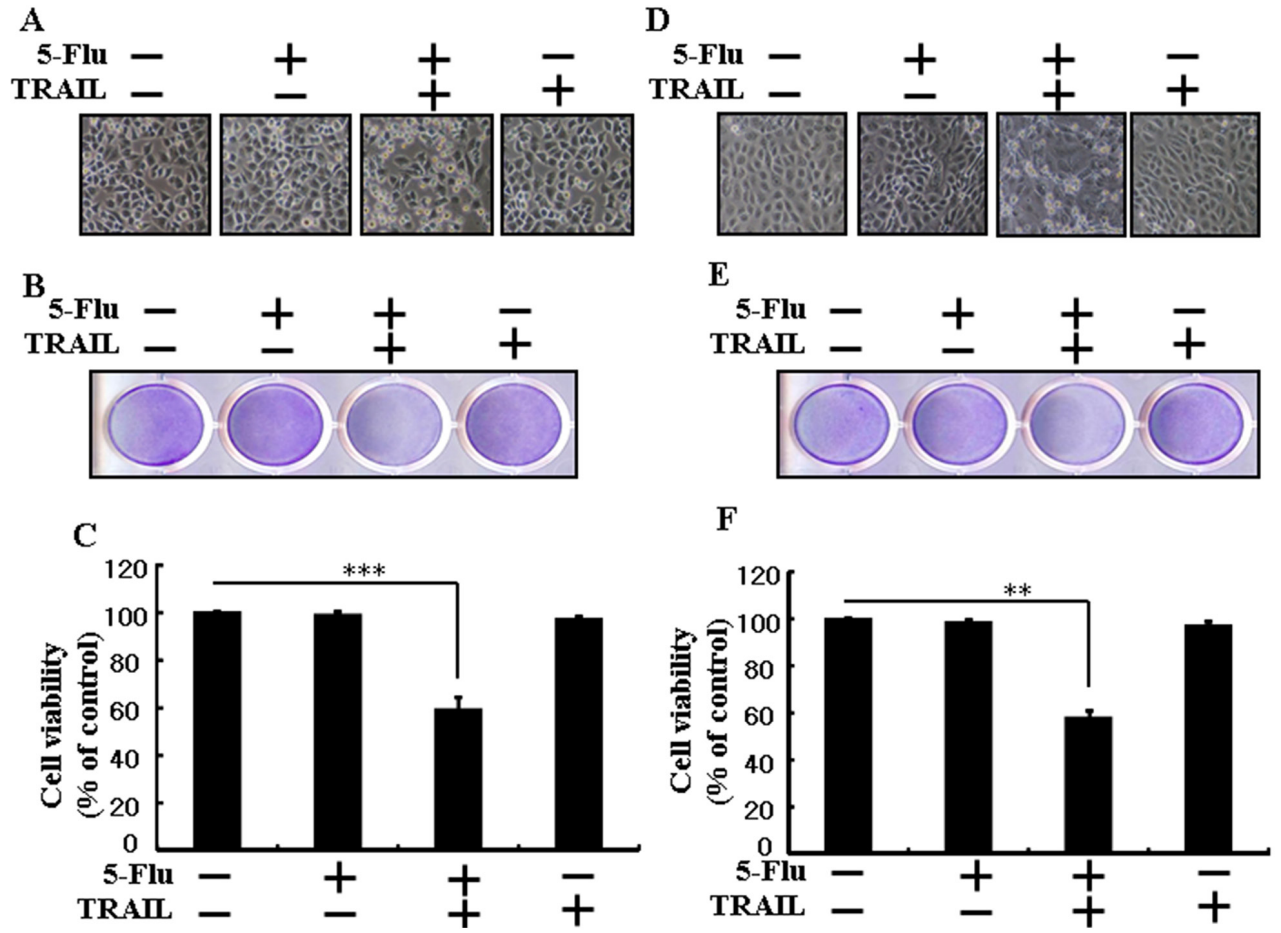
5-Fluorouracil sensitizes TRAIL-induced apoptosis in different types of lung cancer cells HCC-15 and Calu-3 cells were pretreated with 5-fluorouracil at varying concentrations (0, 10, 20, and 40 μM) for 12 h followed by treatment with 200 ng/mL of TRAIL protein for an additional 2 h. **A** and **D**. Cell morphology photographed under light microscope (×100); **B** and **E**. Cell viability determined with crystal violet assay; **C** and **F**. Bar graph showing the average density of crystal violet dye; ** *p* < 0.01, *** *p* < 0.001: significant differences between control and each treatment group; 5-Flu: 5-Fluorouracil; TRAIL: Tumor necrosis factor (TNF)-related apoptosis-inducing ligand.

To determine the effect of 5-fluorouracil on TRAIL-induced apoptosis, HCC-15 and Calu-3 cells were pretreated with different concentrations of 5-fluorouracil for 12 h, followed by treatment with TRAIL for 2 h. Morphological changes in these cells were determined by photographing the cells under a light microscope. 5-Fluorouracil or TRAIL treatment alone did not affect the viability or only slightly affected the viability of HCC-15 and Calu-3 cells (Figure 6). Moreover, TRAIL treatment alone did not induce any morphological changes in HCC-15 and Calu-3 cells compared with those in control cells, indicating that these cells were highly resistant to TRAIL. In contrast, cotreatment of TRAIL with different concentrations of 5-fluorouracil significantly decreased the viability of HCC-15 and Calu-3 cells compared with 5-fluorouracil or TRAIL treatment alone. Results of cell morphology analysis also confirmed this enhanced effect of 5-fluorouracil, indicating that TRAIL and 5-fluorouracil cotreatment increased the apoptosis of cancer cells compared with 5-fluorouracil or TRAIL treatment alone (Figure 1A and 1D). TRAIL and 5-fluorouracil cotreatment decreased the viability and significantly increased the apoptosis of HCC-15 and Calu-3 cells (Figure 1B, 1C, 1E, and 1F). These results indicate that 5-fluorouracil significantly increases TRAIL-induced apoptosis of TRAIL-resistant human lung cancer cell lines HCC-15 and Calu-3.

## DISCUSSION

The present study investigated the function of 5-fluorouracil and the effect of cotreatment with 5-fluorouracil and TRAIL on human lung adenocarcinoma A549 cells. Our results suggest that 5-fluorouracil enhances TRAIL-induced apoptosis of human lung adenocarcinoma A549 cells by increasing Bax and p53 expression.

Chemotherapy is one of the main pathways for treating cancer patients, it is still an ultimatum to increase its anticancer efficacy, diminish side effects and alleviate drug resistance. Use of different drug combinations that target different lanes would significantly improve anticancer capacity, thus potentially offering an effective approach for improving chemotherapy. TRAIL is a promising anticancer substitute because of its outstanding ability to selectively kill tumor cells. TRAIL is a potential chemotherapeutic agent because of its remarkable antitumor activity against various cancer types and because it exerts minimum cytotoxic effects on most of normal cells and tissues [25, 26]. TRAIL induces apoptosis and inhibits the growth of NSCLC in xenograft models [27, 28]. 5-Fluorouracil exerts anticancer effects by metabolically converting to 5-fluorouridine 5’-triphosphate and by subsequently fusing with an RNA and/or by forming 5-fluoro-2’-deoxyuridine 5’-monophosphate, a well-established inhibitor of thymidylate synthetase [29]. 5-Fluorouracil-based combination therapies are used as standard chemotherapy regimens for treating many patients with various malignant tumors, including NSCLC [30–32]. Most anticancer therapeutic agents inhibit cancer progression by directly killing cancer cells through apoptosis [33–36]. Apoptosis can be induced by different stimuli that activate the intrinsic or extrinsic apoptotic pathways. Many DNA-damaging anticancer drugs activate the intrinsic apoptotic pathway, which is negotiated by the damage to the mitochondrial membrane potential and release of cytochrome C and SMAC [37, 38].

Recent evidence indicates that several cancer cell lines, including human lung adenocarcinoma A549 cells, are resistant to TRAIL-induced apoptosis [39]. Results of the present study also showed that 5-fluorouracil or TRAIL treatment alone did not or only slightly induced the apoptosis of human lung adenocarcinoma A549 cells. However, 5-fluorouracil and TRAIL cotreatment significantly induced the apoptosis of human lung adenocarcinoma A549 cells resistant to 5-fluorouracil or TRAIL treatment alone (Figure 1). This suggests that 5-fluorouracil, which exerts anticancer effect when administered along with TRAIL, enhances TRAIL-induced apoptosis of TRAIL-resistant lung adenocarcinoma A549 cells. Results of some studies suggest that 5-fluorouracil treatment inhibits the proliferation and induces the apoptosis of lung cancer A549 cells [40]. However, results of western blotting and ICC performed in the present study showed that 5-fluorouracil and TRAIL cotreatment increased Ac-cas3 and Ac-cas8 expression levels compared with those in control human lung adenocarcinoma A549 cells (Figure 2). This suggests that 5-fluorouracil enhances TRAIL-induced apoptosis of human lung adenocarcinoma A549 cells. Some studies have shown that 5-fluorouracil treatment enhances Bax and p53 expression in Caki-1 cells [17]. Our results suggest that 5-fluorouracil treatment increases Bax and p53 expression in a dose-dependent manner. Our results also indicate that 5-fluorouracil and TRAIL cotreatment increases Bax and p53 expression gradually (Figures 3, 4, and 5).

In conclusion, we observed that 5-fluorouracil induces synergistic apoptosis of A549 cells by increasing the expression of proapoptotic Bax and p53. In addition, our results showed that 5-fluorouracil and TRAIL cotreatment strongly potentiated the apoptosis of various TRAIL-resistant cell lines. Thus, these results suggest that simultaneous administration of 5-fluorouracil and TRAIL can be efficiently used to overcome TRAIL resistance in many tumor types.

## MATERIALS AND METHODS

### Cell culture

Cancer cells originating from lung tumors (A549 cells) were obtained from American type Culture Collection (Global Bioresource Center, Manassas, VA, USA). Bax-containing (Bax^+/+^) and Bax-deficient (Bax^−/−^) HCT116 human colon carcinoma cell lines were provided by Dr. Bert Vogelstein (Johns Hopkins University School of Medicine, Baltimore, MD). All the cells were cultured in RPMI-1640 medium (Gibco BRL, Grand Island, NY, USA) supplemented with 10% (v/v) fetal bovine serum and antibiotics (100 μg/mL penicillin–streptomycin) at 37°C in 5% CO_2_.

### Reagents

Recombinant 5-fluorouracil was purchased from Sigma-Aldrich (St. Louis, MO, USA). TRAIL (200 ng/mL) was purchased from AbFrontier (Geumcheon-gu, Seoul, South Korea).

### Cell viability analysis

A549 cells were plated in 12-well plates at a density of 1.0 × 10^4^ cells and were incubated at 37°C for 24 h. The cells were pretreated with glipizide in a dose-dependent manner (0, 10, 20, and 40 μM). At 12 h after the pretreatment, the cells were treated with 200 ng/mL recombinant TRAIL and were incubated for 2 h. Cell morphology was assessed by examining the cells under an inverted microscope (Nikon, Japan). Cell viability was determined by performing crystal violet staining method as previously described [41].

### Trypan blue dye exclusion assay

The number of viable cells was determined by performing trypan blue dye exclusion assay (Sigma-Aldrich) with a hemocytometer. Results of the dye exclusion assay are expressed as the percentage of viable cells compared to that of vehicle-treated control cells.

### Western blotting analysis

The cells were harvested, washed in cold PBS, resuspended in lysis buffer (25 mM HEPES [pH 7.4], 100 mM EDTA, 5 mM MgCl_2_, 0.1 mM DTT, and protease inhibitor mixture), and sonicated to prepare cell lysates. Proteins (35 μg) present in the cell lysates were separated by performing electrophoresis on 10%–15% SDS polyacrylamide gels and were transferred onto nitrocellulose membranes, and analyzed by western blotting as described previously [42]. Immunoblotting was performed using antibodies against cleaved caspase-3 (Cell Signaling Technology, Danvers, MA, USA), cleaved caspase-8 (BD Pharmingen, USA), Bax and p53 (Santa Cruz Biotechnology, Inc., Santa Cruz, CA, USA), DR4, DR5, and ß-actin (Sigma-Aldrich). Images were obtained using Fusion-FX7 imaging system (Vilber Lourmat, Marne-la-Vallée, France).

### Immunocytochemistry

A549 cells cultured on glass coverslips were treated with 5-fluorouracil and/or TRAIL, washed with PBS, and fixed with 3%–4% paraformaldehyde in PBS at room temperature (RT) for 15 min. The cells were washed twice with ice-cold PBS and were incubated in PBS containing 0.25% Triton X-100 at RT for 10 min. Next, the cells were washed three times with PBS (5 min/wash). After blocking with 1% BSA in PBST for 30 min, the cells were incubated with a primary antibody (anti-cleaved caspase-3 antibody diluted with 1% BSA in PBST) in a humidified chamber at RT for 1 h or at 4°C overnight, followed by washing three times with PBS (5 min/wash). The cells were then incubated in the dark with a secondary antibody (diluted with 1% BSA in PBST) for 1 h at RT. Next, the cells were washed three times with PBS (5 min/wash), incubated with DAPI for 1 min, and rinsed with PBS. Finally, the cells were mounted using a fluorescent mounting medium and were visualized under a fluorescence microscope.

### Statistical analysis

Unpaired *t*-test or Welch's correction was used for comparing between two groups. Multiple comparisons were performed using one-way analysis of variance followed by Tukey–Kramer test. All statistical analyses were performed using GraphPad Prism software. A *p* value of less than 0.05 (*), 0.01 (**), or 0.001 (***) was considered statistically significant.
